# Targeted SPION siderophore conjugate loaded with doxorubicin as a theranostic agent for imaging and treatment of colon carcinoma

**DOI:** 10.1038/s41598-021-92391-w

**Published:** 2021-06-22

**Authors:** Rahim Nosrati, Khalil Abnous, Mona Alibolandi, Jafar Mosafer, Sadegh Dehghani, Seyed Mohammad Taghdisi, Mohammad Ramezani

**Affiliations:** 1 Department of Pharmaceutical Biotechnology, School of Pharmacy, Mashhad University of Medical Sciences, Mashhad, Iran; 2 Pharmaceutical Research Center, Pharmaceutical Technology Institute, Mashhad University of Medical Sciences, Mashhad, Iran; 3 Department of Medicinal Chemistry, School of Pharmacy, Mashhad University of Medical Sciences, Mashhad, Iran; 4 Department of Nanomedicine, School of Paramedical Sciences, Torbat Heydariyeh University of Medical Sciences, Torbat Heydariyeh, Iran; 5 Department of Radiology, 9 Dey Educational Hospital, Torbat Heydariyeh University of Medical Sciences, Torbat Heydariyeh, Iran; 6 Department of Medical Biotechnology and Nanotechnology, Faculty of Medicine, Mashhad University of Medical Sciences, Mashhad, Iran; 7 Targeted Drug Delivery Research Center, Pharmaceutical Technology Institute, Mashhad University of Medical Sciences, Mashhad, Iran

**Keywords:** Cancer imaging, Cancer therapy, Tumour biomarkers, Drug delivery, Drug delivery, Cancer, Nanobiotechnology, Nanomedicine, Diagnostic devices, Drug delivery, Imaging techniques and agents, Nanotechnology in cancer, Nanoparticles, Drug delivery

## Abstract

Recently, the siderophores have opened new horizons in nanomedicine. The current study aimed to design a theranostic platform based on superparamagnetic iron oxide nanoparticles-pyoverdine (SPION/PVD) conjugates bound to MUC1 aptamer (MUC1_Apt_) and loaded with doxorubicin (DOX) as an anti-cancer agent. The SPION/PVD complex was covalently conjugated to MUC1_Apt_ and loaded with DOX to prepare a targeted drug delivery system (SPION/PVD/MUC1_Apt_/DOX). The investigation of cellular cytotoxicity and uptake of formulations by MTT and flow cytometry in both MUC1 positive (C26) and MUC1 negative (CHO) cell lines revealed that MUC1_Apt_ could improve both cellular uptake and toxicity in the C26 cell line. The evaluation of tumor-targeting activity by in vivo bio-distribution showed that the targeted formulation could enhance tumor inhibitory growth effect and survival rate in C26 tumor-bearing mice. Furthermore, the potential of synthesized SPION/PVD/MUC1_Apt_/DOX complex as diagnostic agents was investigated by magnetic resonance imaging (MRI) which improved the contrast of tumor site in MRI. Our findings confirm that aptamer-targeted PVD chelated the SPION as a diagnostic agent and loaded with DOX as a chemotherapeutic drug, would be beneficial as a novel theranostic platform.

## Introduction

Siderophores are low-molecular-weight organic compounds produced by microorganisms for iron uptake in iron deficiency status^1,2^. Based on their iron-binding moieties, siderophores are typically classified as carboxylates, catecholates (or phenolates), and hydroxamates^3,4^. Pyoverdine (PVD) is a mixed-type siderophore with hydroxamate and catecholate groups. PVDs are diffusible yellow-green fluorescent siderophores secreted especially by *Pseudomonas fluorescens*^5^. PVDs are consist of three segment including a fluorescent dihydroxyquinoline-type chromophore with a quenching response to Fe(III), an acyl side chain (either amide or dicarboxylic acid) bound to the chromophore via its NH_2_-group; and a variable peptide chain with 6 to 12 amino acids attached via its amide group to the carboxyl group of the chromophore^5,6^. The strong binding of PVDs-Fe has a very high stability constant of approximately 10^22^ to 10^32^ M^−1^ which protects them from hydrolysis and enzymatic degradation^7^.

Recently, the medical application of siderophores has drawn much attention^1^. Siderophores are used in removal of transuranic elements from the body, the treatment of iron overload diseases (for example sickle cell anemia), antimalarial activity^8^, molecular imaging^9^ and cancer therapeutics^10^. In drug delivery systems, siderophores have been used as “Trojan horse strategy” for the careful transfer of antibiotics into antibiotic-resistant bacteria^11,12^. In the cancer treatment approach, siderophores can reduce the access of cancer cells to Fe^1^. The superparamagnetic iron oxide NPs (SPIONs)-siderophore conjugates can also be used as tumor imaging probes for magnetic resonance imaging (MRI)^1^.

Aptamers as a kind of targeting molecules are single-stranded nucleic acids with high affinity and selectivity against the cancer markers^13,14^. Mucine 1 aptamer (anti-MUC1; MUC1_Apt_), one of the most studied aptamers, can specifically recognize mucin 1 (MUC1) protein. MUC1 is a membrane-associated heterodimeric glycosylated protein formed by two subunits and expressed on the apical surface of most normal epithelial cells^15^. A type of MUC1 that is aberrantly overexpressed on the majority of cancer cell surfaces, such as human colon cancer, is commonly mentioned as tumor-associated MUC1 (TA-MUC1) which differs from its normal type in several properties^16,17^. These structural differences, have made TA-MUC1 an attractive target for specific diagnosis and treatment of cancers^18,19^.

Aptamer-mediated targeted delivery of the chemotherapeutic agents to cancer cells can enhance the drive of an anti-cancer drug to target cells, increase the residence time of the therapeutics at the tumor site, and reduce the healthy tissues toxicity^20,21^. Among chemotherapeutics, doxorubicin (DOX) is one of the most used aptamer-drug bio-conjugates in various approaches of cancer therapy^22^. DOX is directly intercalated into the CG sequence of the aptamer and provides efficient and specific delivery into target cancer cells^21,23^. This platform overcomes the limitations of free DOX chemotherapy such as the DOX distribution to normal healthy tissues and consequent systemic toxicity^21,22^.

**Figure 1:**
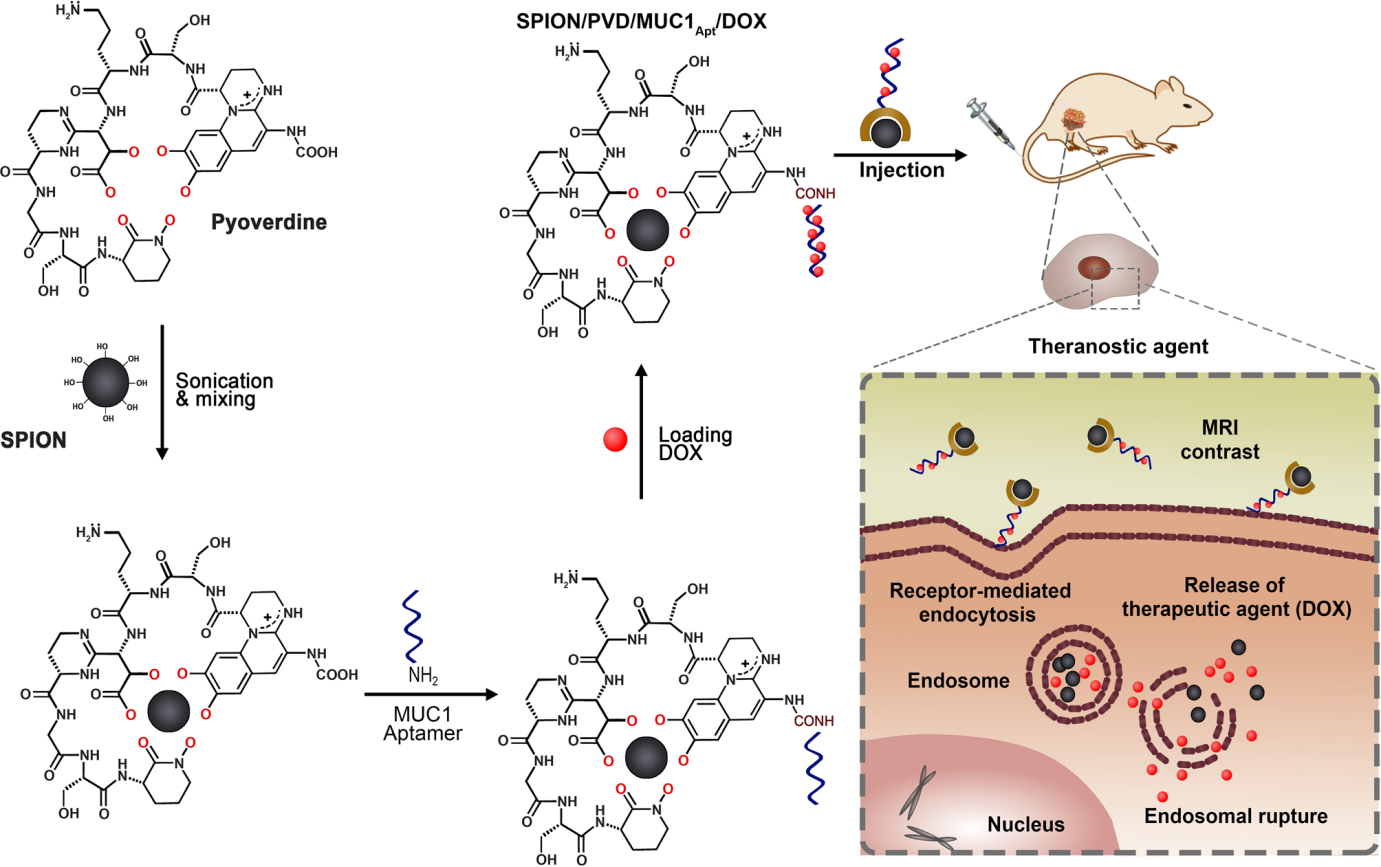
Schematic illustration of MUC1_Apt_-based targeted system for delivery of SPION/PVD loaded with DOX.

The combination of SPIONs-siderophore conjugate with MUC1_Apt_ as a targeting molecule and DOX as a chemotherapeutic drug, can provide a proper theranostic agent for the integration of tumor diagnosis and targeted anti-cancer drug delivery. So, in this study, the purified bacterial PVD was conjugated to synthesized SPIONs. Then, SPION/PVD was covalently conjugated to MUC1_Apt_ and loaded with DOX to fabricate a theranostic platform (Fig. 1). In addition to in vitro drug loading capacity and release pattern of DOX, the cellular uptake and cytotoxicity effects of formulations were also investigated. In order to investigate the potential of synthesized NPs as diagnostic agents, the MRI technique was carried out. Furthermore, the tumor-targeting activity of formulations was evaluated by in vivo bio-distribution study on mice bearing C26 colon carcinoma.

## Results

### Characterization of purified pyoverdine

**Figure 2:**
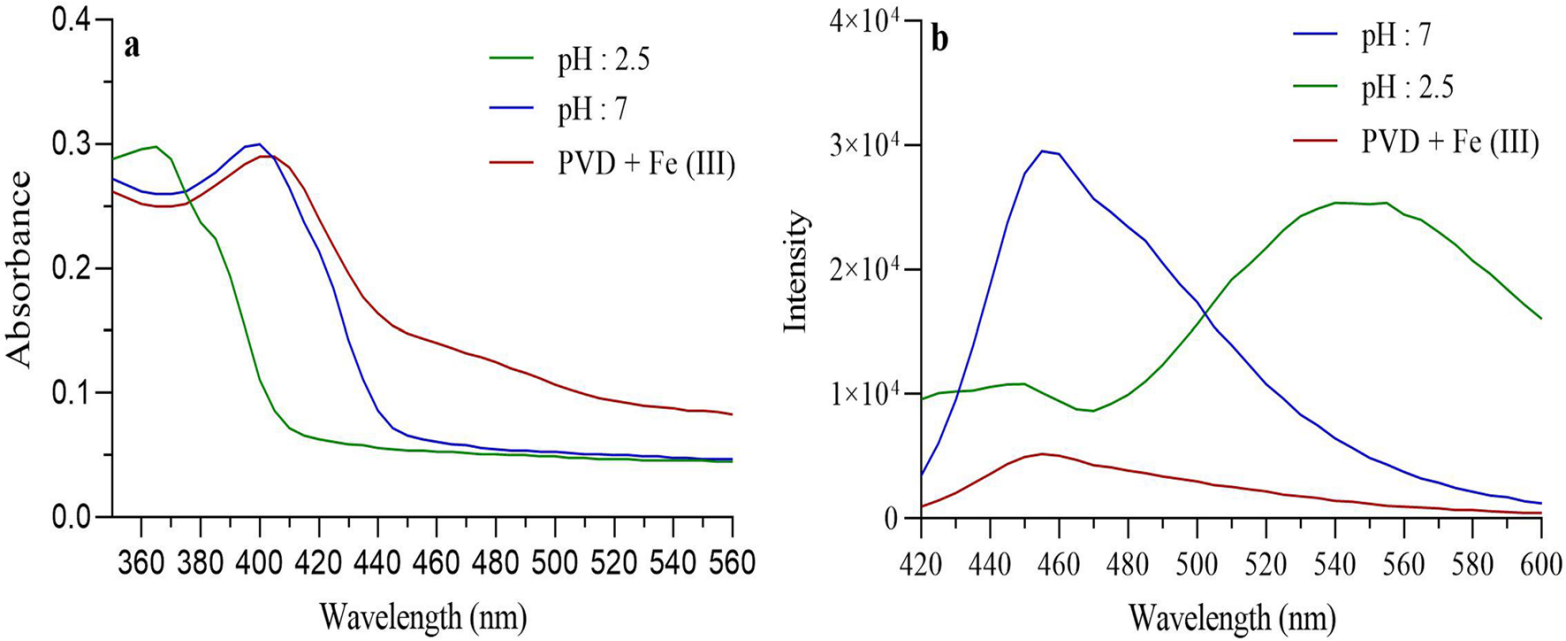
Characterization of the purified PVD from *P. fluorescens* IBRC-M 10752 strain using (**a**) UV absorption spectroscopy (*λ*_max_: 400 nm); and (**b**) fluorescence emission at excitation 400 nm.

The obtained PVD was characterized by several methods. Based on the absorption spectrum of PVD solution, the maximum peak was obtained at *λ*_400_ (pH 7) and 360 nm (pH 2.5) while the largest peak of the Fe-PVD complexes shifted to 405 nm with broad charge transfer bands at about 470 to 550 nm (Fig. 2a). PVD was also identified by its known fluorescence; excitation at 400 nm and emission read at 460 nm (pH 7) and 550 nm (pH 2.5) (Fig. 2b). The positive results for Csaky’s test (producing of deep red color) and Arnow’s assay (the presence of pink color) revealed the presence of a siderophore with catecholate and hydroxamate functional groups. The efficiency of PVD fluorescence quenching in the presence of FeCl_3_ solution showed that fluorescence intensity decreased gradually with increase in the concentration of Fe ions (Fig. 2b). The analysis of extracted pigment using the TLC with solvent system butanol:acetic acid:water (3:1:1) as mobile phase was indicated a single fluorescence spot with an R_*f*_ value of 0.8 for purified siderophore. The molecular weight of purified PVD obtained by LC/MS/MS spectroscopy was about 1364 Da, which was similar to the previous reports^24^. The concentration of PVD was calculated to be 360 µmol/L.

### Characterization of synthesized SPIONs and SPION/PVD conjugates

**Figure 3:**
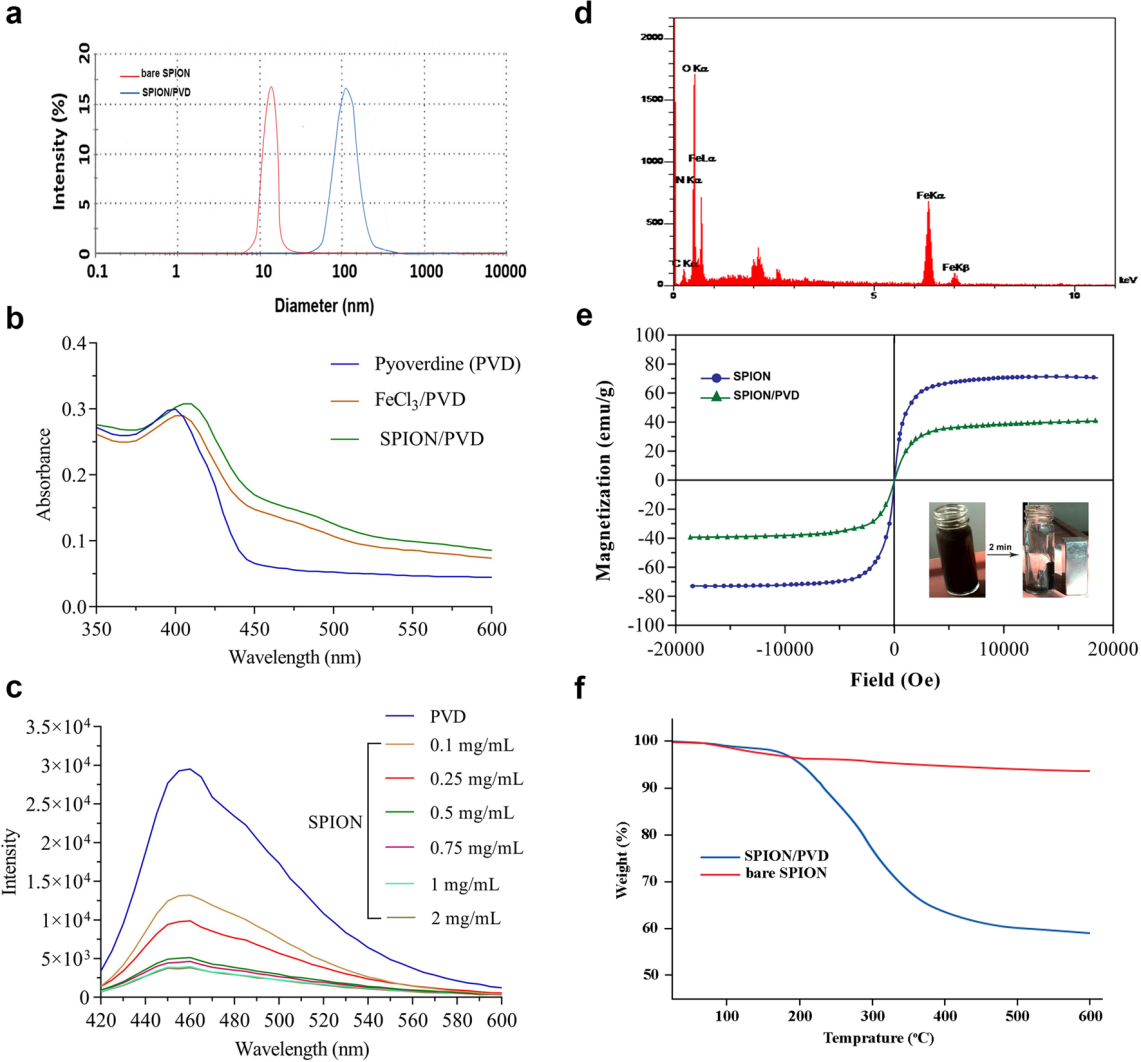
(**a**) Size distribution of the bare SPION and SPION/PVD conjugates, (**b**) UV absorption spectroscopy, (**c**) Fluorescence emission at excitation 400 nm, (**d**) EDX patterns, (**e**) VSM magnetization curves of SPION versus SPION/PVD and the directed movement under an external magnetic field, and (**f**) comparison between the TGA profiles of SPION and SPION/PVD.

The intensity size distribution from DLS measurements (Fig. 3a) showed that the bare SPION and SPION/PVD formulation had a mean hydrodynamic diameter around 27.9 ± 4.7 and 96.5 ± 7.1 nm with 20.1 ± 1.7 and − 1.3 ± 0.1 mV zeta potentials, respectively (Table 1). The SEM images of bare SPION and SPION/PVD confirmed the unimodal spherical shape and smooth surface with a size around 20 nm and 100 nm, respectively (see Supplementary Fig. S1 online). Similar to Fe-PVD, the conjugation of SPION to PVD caused a shift in the maximum absorption to 405 nm with broad charge transfer bands around at 470–550 nm in comparison with PVD spectrum (Fig. 3b). The fluorescence quenching efficiency of PVD in the presence of SPION (0.1, 0.25, 0.5, 0.75, 1, and 2 mg/mL) showed that the fluorescence intensity decreased gradually with increasing of the concentration of SPION (Fig. 3c). Elemental signals of C, N, Fe and O were also detected in the EDX spectrum confirming the successful conjugation of PVD to SPION (Fig. 3d). Based on VSM results, the saturation magnetization (M*s*) values of SPION and SPION/PVD at room temperature were 72.7 emu/g and 36.9 emu/g, respectively (Fig. 3e). The results of the TGA indicated two obvious slopes for bare SPION and SPION/PVD. The firsts slope emerged in the range of 100–170 °C was ascribed to the release of hydration water in the complex (~ 1.5%). In bare SPION, another visible slope between 200 and 600 °C was observed, which can be attributed to the removal of OH group. The total weight loss for SPION was calculated as 7.3%. After that, the PVD conjugated to SPION started to decompose slowly from 210 to 600 °C and the weight was reduced, thus it could be concluded that the content of PVD in SPION/PVD complex was about ~ 34% (Fig. 3f).

**Table 1:** DLS analysis of the prepared formulation.

Formulation	Size (nm)	PDI	Zeta Potential (mV)
Bare SPION	27.9 ± 4.7	0.31 ± 0.05	20.1 ± 1.7
SPION/PVD	96.5 ± 7.1	0.21 ± 0.08	− 1.3 ± 0.1
SPION/PVD/MUC1_Apt_	119.8 ± 4.6	0.25 ± 0.04	− 10.2 ± 1.2
SPION/PVD/MUC1_Apt_/DOX	127.6 ± 5.8	0.38 ± 0.01	− 4.33 ± 0.7

### FTIR analysis of PVD and SPION/PVD conjugates

FTIR analysis showed the presence of the main functional groups in PVD (see Supplementary Fig. S2a online). The broad absorption at ~ 3225.4 cm^−1^ has contributions from O–H and N–H stretching while the peak at 2404.1 cm^−1^ corresponds to NH_3_ overtone. The peaks at 1664 and 1268 cm^−1^ are attributed to C=O and C–O stretching modes in C=O moiety of amide group (O=C–NH_2_) and phenolic group, respectively. The peak at 1082.6 cm^−1^ has possibly some contributions from C–O–C bending. The absorption at 868.74 cm^−1^ is attributed to the CH plane binding in the aromatic ring. In the spectrum of SPION/PVD, the C=O stretch band at 1664 cm^−1^ disappeared whereas two new bands at 1402 and 1630 cm^−1^ observed, confirming the prominent absorptions related to the amide I and II modes of the PVD (see Supplementary Fig. S2b online). Also, the CH_2_ bands at 2924 (asymmetric stretching) and 2851 cm^−1^ (symmetric stretching) have shifted to a lower frequency wavelength suggesting a rearrangement of the hydrocarbon molecules surrounding the NPs. Peak corresponding to the O–H group on the catechol-like group of PVD-chromophore changed to 3414 cm^−1^ indicating a bidentate covalent bond with Fe. The bands at 1402 and 3124 cm^−1^ show the major contributions of bending vibrations of amides and N–H stretching on the peptide chain bonded to SPION. The C–O stretching frequency for PVD at 1088 cm^−1^ is shifted to 1025 cm^−1^, which further confirmed that the 2, 3-diamino 6, 7-dihydroxyquinoline group of the PVD binds covalently to the Fe_3_O_4_. The band at 566 cm^−1^ corresponding to Fe–O stretching shows the formation of Fe–O coordinate bond at Fe_3_O_4_. These results confirmed the formation of the PVD-chelated Fe_3_O_4_ NPs.

### Aptamer conjugation

In DLS analysis, an increase about ~ 19 nm in the size of the prepared complex was obtained after the conjugation of MUC1_Apt_ to SPION/PVD suggesting the aptamer conjugation with the complex. Due to the negative charge of Aptamer, the zeta potential of the aptamer-conjugated SPION/PVD was lower than that of SPION/PVD (Table 1). Results of gel retardation assay indicated that SPION/PVD/MUC1_Apt_ complex (Lane 2) was unable to penetrate through the pores in the gel, demonstrating the high molecular weight of SPION/PVD/MUC1_Apt_ complex compared to free MUC1_Apt_ (Lane 3) which was significantly shifted (see Supplementary Fig. S3 online). Also, no band was observed for SPION/PVD (lane 1). The covalent conjugation of MUC1 aptamer to the SPION/PVD complex was confirmed by treating the SPION/PVD/MUC1_Apt_ with DTT as the reducing agent (Fig. S3b). A band corresponding to free aptamer appeared indicating the cleavage of the aptamer from the SPION/PVD/MUC1_Apt_ conjugate.

### In vitro drug loading and release

**Figure 4:**
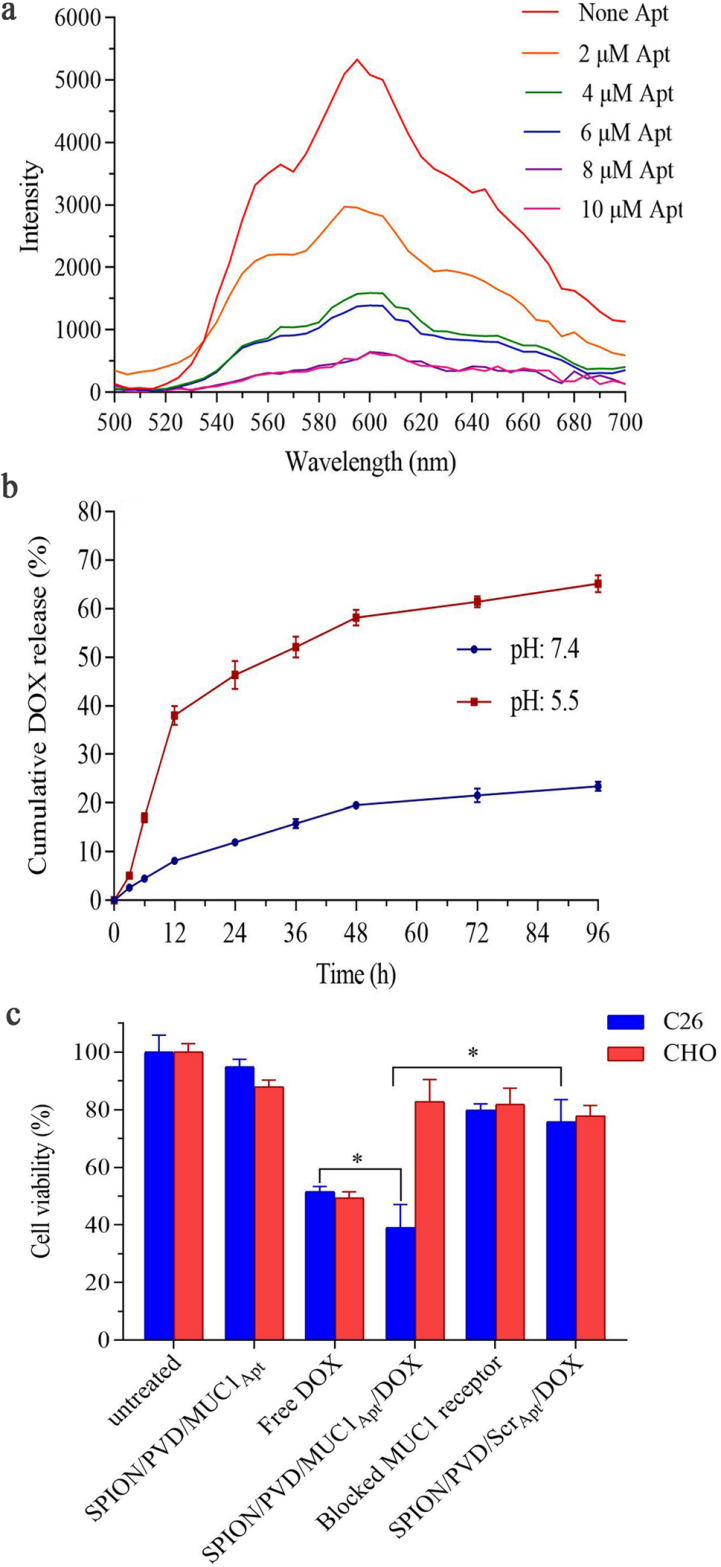
(**a**) Fluorescence spectra of DOX (2 µM) after treatment with increasing concentrations of SPION/PVD/MUC1_Apt_ complex (based on aptamer concentration 0, 2, 4, 6, 8, and 10 µM); (**b**) In vitro DOX release profile of SPION/PVD/MUC1_Apt_/DOX in citrate buffer (pH 5.5) and PBS (pH 7.4) at 37 °C; (**c**) Cell viability assessments of free DOX, SPION/PVD/MUC1_Apt_, SPION/PVD/MUC1_Apt_/DOX and SPION/PVD/Scr_Apt_/DOX in C26 and CHO cell lines after 48 h of incubation at 37 °C. Data are shown as mean ± SD (n = 3). Statistical analysis was performed using GraphPad Prism Version 8.0 (https://www.graphpad.com).

To confirm the intercalation of DOX molecules into SPION/PVD/MUC1_Apt_, the fluorescent intensity of DOX was investigated after the addition of DOX to SPION/PVD/MUC1_Apt_ with different content of aptamer. At 4:1 ratio of the aptamer:DOX (8 µM:2 µM), the maximum reduction of the fluorescent intensity of DOX was observed (Fig. 4a). The entrapment efficiency (EE) was ~ 92% with loading capacity (LC) of 6.18%. The size analysis of SPION/PVD/MUC1_Apt_ by DLS revealed that an increase from 119.8 ± 4.6 to 127.6 ± 5.8 nm verifying the loading of DOX (Table 1). Moreover, the change of zeta potential of SPION/PVD/MUC1_Apt_ from − 10.2 to − 4.33 mV displayed the loading of DOX as a cationic molecule (Table 1). The SEM images also confirmed that the SPION/PVD/MUC1_Apt_/DOX complex has < 200 nm diameter with spherical structure which can be sutible in intravenous delivery (see Supplementary Fig. S1 online).

Figure 4b shows the release rate of DOX in citrate buffer (pH 5.5) and PBS (pH 7.4). When the pH value reduced from 7.4 to 5.5, the content of DOX released from SPION/PVD/MUC1_Apt_/DOX increased significantly (*p ≤ 0.05). DOX represented a constant release at pH 7.4 and only 23% of DOX could be released after 96 h, demonstrating the stability of SPION/PVD/MUC1_Apt_/DOX at biological pH. However, a fast release was recognized at pH 5.5, about ~ 40% in the initial 12 h and then it progressed at a slower rate. After 96 h, 65% of DOX was released from SPION/PVD/MUC1_Apt_/DOX at pH 5.5. The release data showed good correlation with Zero order kinetic, an ideal model for release of drug from the developed formulation, at pH 7.4, While a burst release kinetic was observed as the pH shifted from neutral to acidic.

### Cell viability assay

In vitro cytotoxicity tests was performed to estimate the cytotoxicity of free DOX, SPION/PVD/MUC1_Apt_, SPION/PVD/MUC1_Apt_/DOX and SPION/PVD/Scr_Apt_/DOX in MUC1 positive C26 cell line and CHO cell line lacking MUC1. The results demonstrated that targeted complexes are notably more cytotoxic than free DOX and SPION/PVD/Scr_Apt_/DOX as a non-targeted complex in C26 cells (p ≤ 0.05). Moreover, compared to SPION/PVD/MUC1_Apt_/DOX-treated C26 cells, the cell viability of blocked-MUC1 receptor on C26 cells after treatment with SPION/PVD/MUC1_Apt_/DOX improved, indicating the prevention of MUC1_Apt_ attachment to the target cells. However, in CHO cell line, the targeted MUC1-modified complex and non-targeted scramble aptamer-modified complex was not shown significant difference in term of cytotoxicity (*p > 0.05) (Fig. 4c).

### Cellular uptake analysis

**Figure 5:**
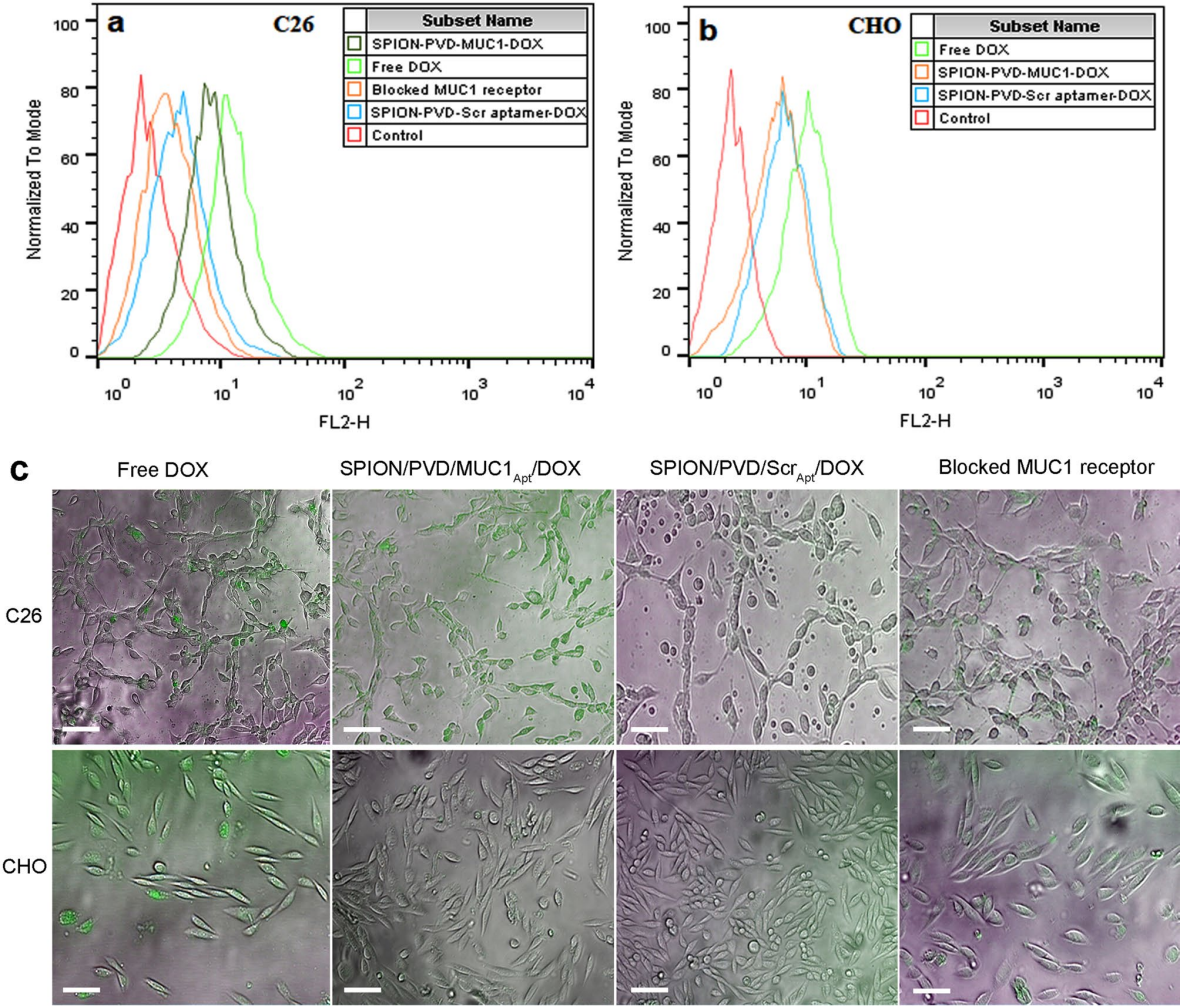
Quantitative and qualitative analysis of internalization of free DOX, SPION/PVD/MUC1_Apt_/DOX and SPION/PVD/Scr_Apt_/DOX into C26 and CHO cell lines after 2.5 h of incubation using (**a**,**b**) flow cytometry and (**c**) fluorescence microscopy (the merged images of the DOX fluorescence channels and bright field; Scale-bar Lengths: 50 μm).

The cellular uptake ability of free DOX, SPION/PVD/MUC1_Apt_/DOX and SPION/PVD/Scr_Apt_/DOX in CHO and C26 cell lines was evaluated by means of flow cytometry. As revealed in Fig. 5a,b, cellular uptake of MUC1_Apt_ targeted-complex was higher than that of scramble aptamer-modified complex (non-target) in C26 as MUC1 positive cells. Based on the competition assay, the pre-mixing of MUC1 receptors with an excess amount of free MUC1_Apt_, reduced the cellular internalization level of with SPION/PVD/MUC1_Apt_/DOX. This phenomenon demonstrated that MUC1_Apt_ could induce receptor-mediated endocytosis of the SPION/PVD/MUC1_Apt_/DOX system in MUC1-positive cell lines. However, no significant difference was observed between CHO treated with SPION/PVD/MUC1_Apt_/DOX and SPION/PVD/Scr_Apt_/DOX (Fig. 5b).

Moreover, fluorescence microscopy images visibly revealed that SPION/PVD/MUC1_Apt_/DOX was mainly accumulated within the C26 cells (Fig. 5c). Pre-treatment of C26 cells with an excess amount of free MUC1_Apt_ decreased the fluoresce intensity of SPION/PVD/MUC1_Apt_/DOX-treated C26 cells, indicating the specific binding and penetration of SPION/PVD/MUC1_Apt_/DOX.

### Magnetic resonance imaging

**Figure 6:**
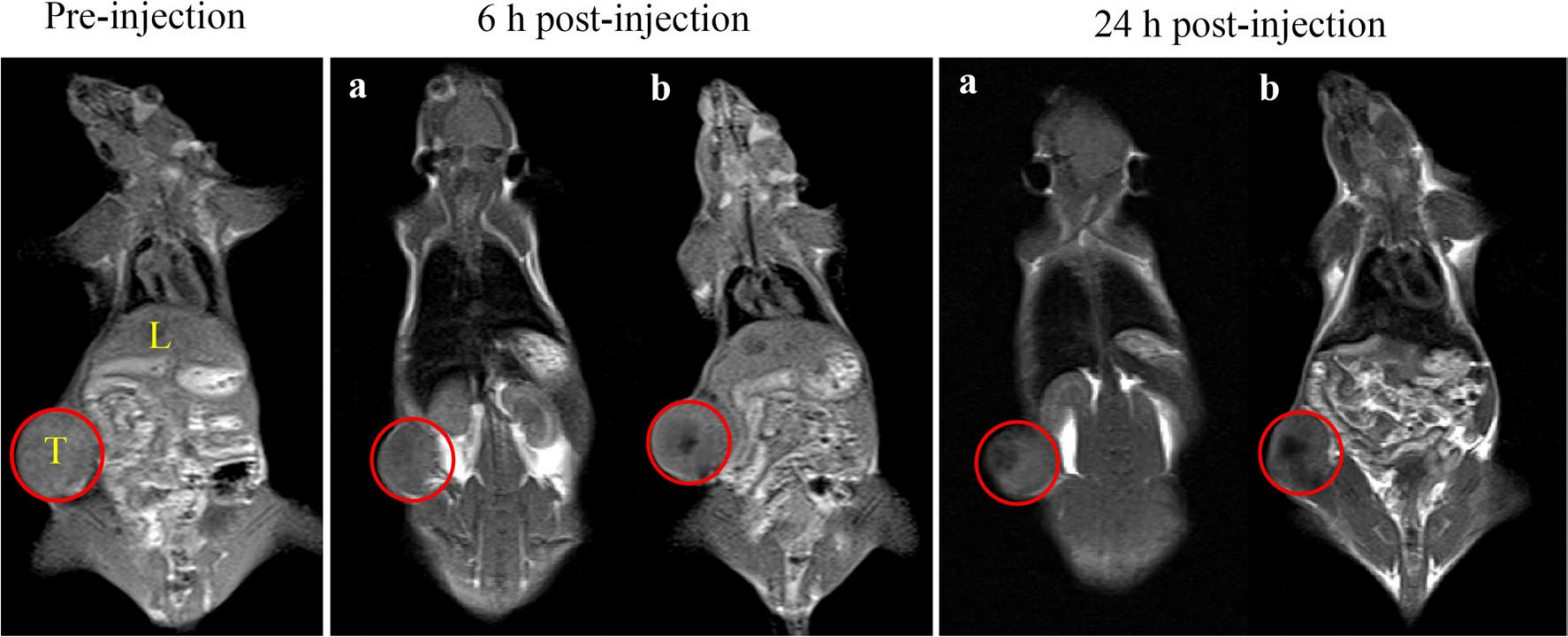
T1-weighted MR images of C26 tumor-bearing male BALB/c mice 6 and 24 h post-injection of (**a**) SPION/PVD and (**b**) SPION/PVD/MUC1_Apt_/DOX (SPION equivalent concentration 0.6 mg/mL).

MRI was showed the magnetic capability of the prepared complexes in C26 tumor-bearing BALB/c mice. T1-weighted MRI after 6 and 24 h post-injection of SPION/PVD and targeted formulations (Fig. 6) revealed that in mice receiving SPION/PVD, even after 6 h post-injection, a clear contrast was not observed in comparison with those in control group and contrast after 24 h was low. In comparison with SPION/PVD, a significant accumulation of SPION/PVD/MUC1_Apt_/DOX was observed in tumor sites. The clear contrast in tumor tissue after 24 h post-injection arose from the ligand-receptor interaction, corroborated the MUC1_Apt_ targeting capability and the prolonged residence times in tumor.

### Ex vivo fluorescence imaging

**Figure 7:**
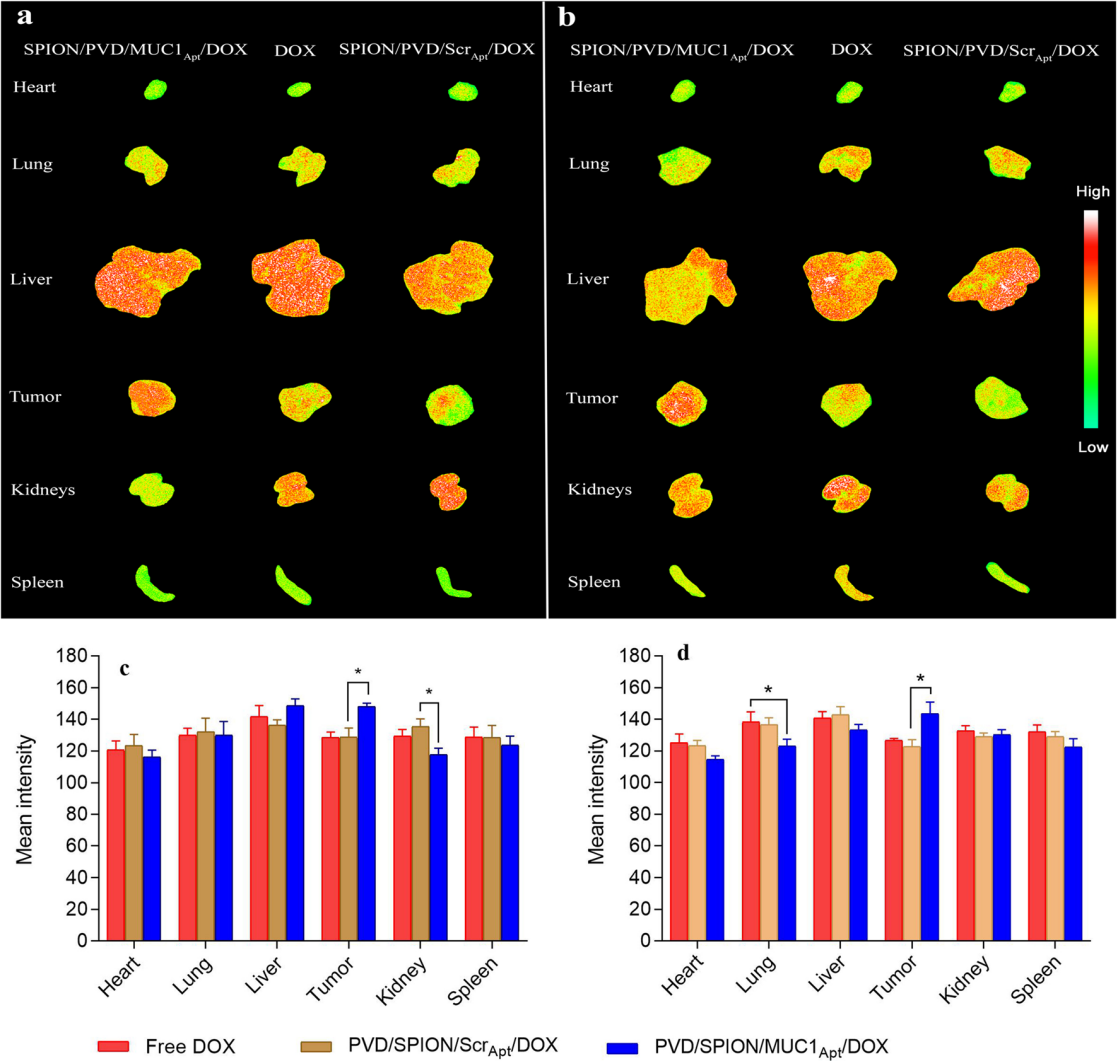
The ex vivo fluorescence images and ROI analysis of tumor and major organs (liver, spleen, kidneys, heart, and lungs) of C26 tumor-bearing BALB/c, (**a**,**c**) 6 h and (**b**,**d**) 24 h post-injection of SPION/PVD/MUC1_Apt_/DOX, SPION/PVD/Scr_Apt_/DOX and free DOX (with equivalent DOX concentration: 0.2 mg/kg). Statistical analysis of parts c and d was performed using GraphPad Prism Version 8.0 (https://www.graphpad.com).

A real-time fluorescence imaging technique was used to check the tumor accumulation and biodistribution of free DOX, SPION/PVD/MUC1_Apt_/DOX and SPION/PVD/Scr_Apt_/DOX in mice bearing C26 tumor 6 and 24 h post-injection (Fig. 7a,b). As shown in Fig. 7, the highest fluorescence intensity in the tumor was observed in mice treated with SPION/PVD/MUC1_Apt_/DOX in comparison with those treated with SPION/PVD/Scr_Apt_/DOX and free DOX, at both 6 and 24 h post-administration, showing the role of MUC1 aptamer in accumulation of the targeted complex in the tumor tissue. This qualitative fluorescence date at tumor site, was confirmed by the quantitative results obtaining by the region of interest (ROI) analysis (Fig. 7c,d). ROI analysis also exhibited that the mean intensity of the targeted formulation in the tumor was significantly higher than that of non-targeted formulations (*p ≤ 0.05). The liver accumulation after 6 h post-injection was high for all groups while SPION/PVD/MUC1_Apt_/DOX injected group proved rapid liver clearance 24 h post-injection. Beside, due to the attachment of targeted formulation to specific receptor in tumor, their leakage from tumor to blood was reduced, leading to low accumulation and low intensity in liver after 24 h in comparison with SPION/PVD/Scr_Apt_/DOX-treated group. Moreover, as it is shown in Fig. 7b,d, in heart and lung tissues of mice treated with SPION/PVD/MUC1_Apt_/DOX at 24 h post-administration, lower intensity of fluorescence was detected in comparison with free DOX and non-targeted formulation.

### In vivo therapeutic potency evaluation

**Figure 8:**
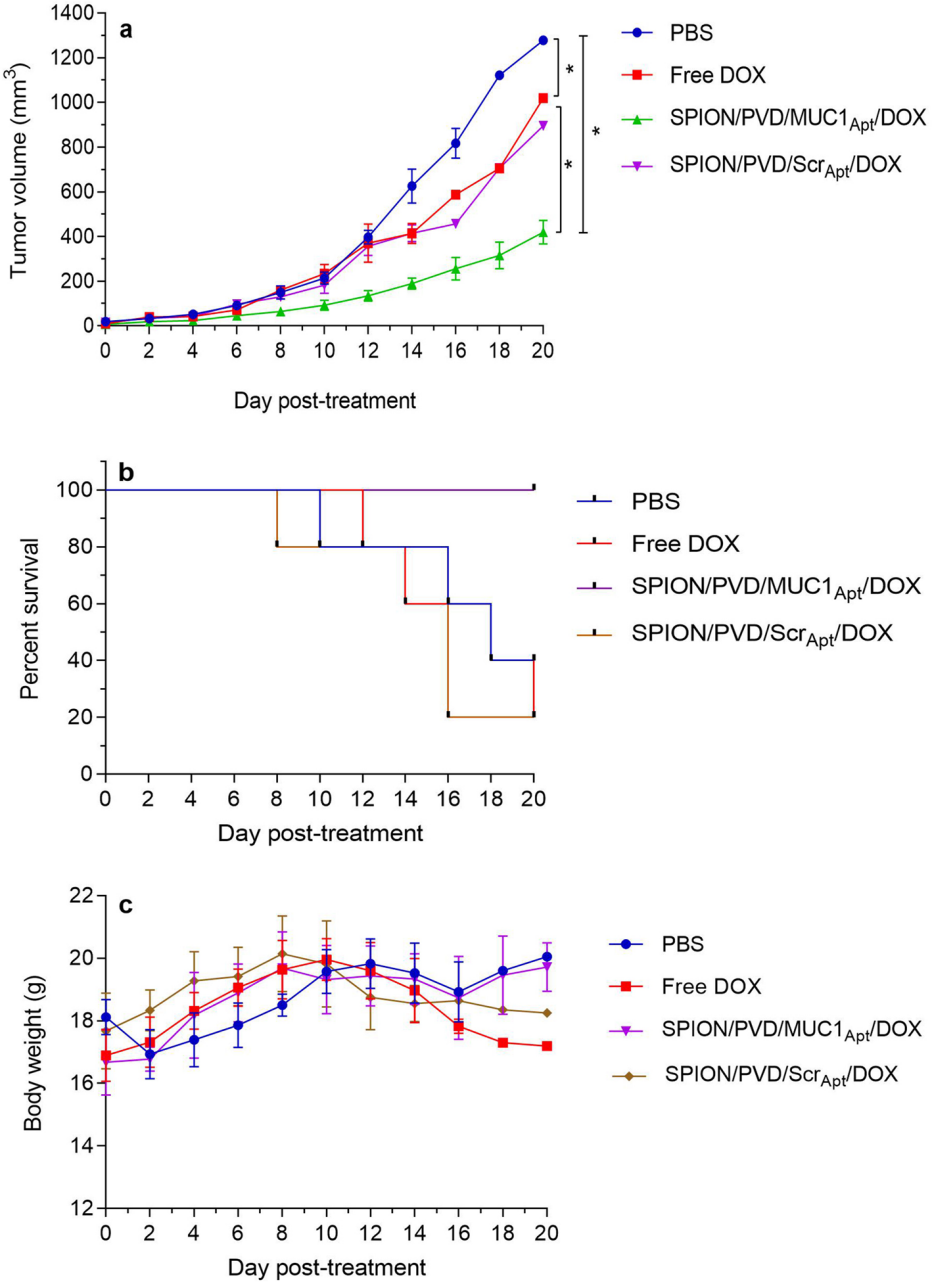
Tumor growth-inhibitory impact (**a**), Kaplan–Meier survival curve of the mice (**b**) and body weight of tumor-bearing mice (**c**) of SPION/PVD/MUC1_Apt_/DOX compared with PBS (control), free DOX, and SPION/PVD/Scr_Apt_/DOX groups (n = 5, error bars represents SD). Statistical analysis was performed using GraphPad Prism Version 8.0 (https://www.graphpad.com).

The therapeutic capability of the prepared nanoformulations was determined by assessing survival time and tumor growth rate of C26 tumor-bearing BALB/c mice after single-dose intravenous injection of free DOX, SPION/PVD/MUC1_Apt_/DOX and SPION/PVD/Scr_Apt_/DOX (equivalent DOX concentration 0.2 mg/kg). The obtained date revealed that the inhibitory effect of SPION/PVD/MUC1_Apt_/DOX, as a targeted-formulation, was remarkably higher than those of PBS, free DOX, and SPION/PVD/Scr_Apt_/DOX (Fig. 8a). No significant difference was also obtained in tumor size between groups receiving free DOX and SPION/PVD/Scr_Apt_/DOX (*p > 0.05). The survival rate results confirmed high survival rate for SPION/PVD/MUC1_Apt_/DOX receiving group compared to other treated groups (Fig. 8b). In addition, the body weight loss in free DOX-receiving mice was greater than SPION/PVD/MUC1_Apt_/DOX-received mice (Fig. 8c).

## Discussion

In summary, we provided a MUC1_Apt_-based targeted system for the delivery of DOX-loaded SPION/PVD (SPION/PVD/MUC1_Apt_/DOX), capable of providing MRI images and preventing cancer cell growth in vitro and in vivo. As far as we know, this is the first study to provide a SPION/PVD conjugate as a MRI diagnostic agent which is targeted with an aptamer and loaded with an anti-cancer drug, providing a theranostic platform.

Due to the aggregation and the instability of bare Fe_3_O_4_ NPs at physiological pH, numerous surface coatings have been used to modify their surface properties and improve the stability^25^. Our results also indicated that while bare SPION is not stable, but PVD/SPIONs/MUC1_Apt_/DOX is stable enough to be tested in vivo (see Supplementary Table S1 online). Besides, the bare SPIONs can be degraded via the body metabolism causing an overload of iron ions in tissues. Inspired by reports that used Fe_3_O_4_ NPs to selectively adsorb siderophores as a microbial chelator with high affinity^26,27^, we demonstrated the capability of magnetic NPs chelated with purified bacterial PVD. Previous studies revealed that SPION can be conjugated with any drugs and/or natural compounds containing sulfhydryl (-SH), amine (-NH_2_), phosphate (PO_4_^3-^), hydroxyl (-OH), and carboxyl (-COOH) groups or their combinations^28,29^. Therefore, hydroxamate and catecholate functional groups of PVD can contribute to the SPION chelation with PVD. It is well-known that the PVD can efficiently bind to metal ions such as Fe, Cu, Zn via the cooperation of dihydroxyquinoline and peptide chain^30^. As PVD chelates iron ion with a 1:1 (PVD:Fe^3+^) stoichiometry^1^, so PVD bound to SPION with a stoichiometry of 1:1. The formation of the Fe(III)-PVD gives rise to a shift in the λ_max_ of the free PVD absorption spectrum and further quench the fluorescence of PVD through electron-transfer pathways (Fig. 2a,b)^31^. In our research, the SPION could be also captured by the hydroxyl and carbonyl groups on PVD and the electron transfer occurred between PVD and the SPION, which further resulted in the λ_max_ shifting and the fluorescence quenching of PVD (Fig. 3). An insight into the possibility of SPION/PVD formation was also confirmed by the FTIR (Fig. S2) which was in agreement with previous reports^27,30,32^. However, SPION/PVD conjugates were previously used as analytical platforms. The binding ability of SPION to PVD indicates that it has more potential for efficient isolation of siderophores from microorganisms media based on magnetic properties of the SPION.

Conjugation of SPION with tumor targeting moieties such as aptamers and loaded with anti-cancer drug represent a promising platform for the efficient capture of cancer biomarker and specific delivery of MRI agents and drugs. Herein, the DOX release pattern from SPION/PVD/MUC1_Apt_/DOX formulation was compared at pH 5.5 and 7.4 to mimicking the tumor cell endosome and physiological conditions, respectively, and the accelerated release rate of DOX was observed at pH 5.5 in comparison with pH 7.4 (Fig. 4b). The accelerated release in the acidic state might be owing to the superior dissolution of the protonated DOX at low pH which also enhanced its water solubility^33^. It is notable to mention that the faster release of anti-cancer drug from our complexes under slightly acidic conditions, similar to pH of the tumor cells endosome, facilitates the therapeutic performance of the formulation and thus improving cellular uptake and cytotoxicity at the site of action^34^. Thus the synthesized SPION/PVD/MUC1_Apt_/DOX complex provides an ideal drug carrier with a pH-responsive trait which can control the release of intercalated DOX. The evaluation of cytotoxicity of formulations on C26 and CHO cell lines (MTT assay) indicated the validity of MUC1_Apt_ on the uptake and cell killing effect of the targeted formulation. This is in agreement with former studies in which delivery of DOX with aptamers like MUC1_Apt_, increased the cytotoxicity and cellular internalization of DOX in vitro^33,35^. The mechanism of aptamer-based drug uptake might be receptor-mediated endocytosis (RME) leading to effective internalization of formulation into the cells with expressed receptor^36,37^.

The high dose infusion, adverse effects, low bioavailability, low therapeutic index, and non-specific targeting are some of the major drawbacks of chemotherapy^38,39^. In this line, the nonselective distribution of DOX in the normal organs leads to severe systemic toxicity especially cardiotoxicity, which limits localization of free DOX in target tumor site^40,41^. In previous attempts to overcome these limitations, the nano-sized aptamer-targeted systems loaded with DOX could improve the biodistribution and antitumor efficacy compared with free DOX^42,43^. It has been also demonstrated that the GC base pairs of the aptamer afford proper places for DOX loading^21,23^. The structure of MUC1_Apt_ was demonstrated to intercalate 2–3 molecules of DOX into the GC sequence of MUC1_Apt_. Using this approach, we intercalated DOX into a MUC1 aptamer conjugated to SPION/PVD (Fig. 4a). In this regard, the intercalation phenomenon of DOX into aptamer structure did not affected the delivery of other components of the formulation (SPION and PVD). In vivo experiments indicated that targeted DOX-loaded complex could simultaneously deliver DOX and SPION to tumor site and increased the accumulation of DOX and SPION in tumor tissues and obviously decreased the their accumulation in normal tissues particularly heart and lungs (Fig. 7). The targeted formulation could also be accumulated in liver 6 h post-injection, probably due to the colloidal nature of non-sized formulation. However, after 24 h, the SPION/PVD/MUC1_Apt_/DOX formulation was less accumulated in liver compared to other treatments because of its targeting characteristics, leading to its high accumulation in tumor tissue. Meanwhile, the greater loss of body weight in free DOX-administrated mice in comparison with mice receiving SPION/PVD/MUC1_Apt_/DOX (Fig. 8c) could be related to the easily circulation of DOX within the body and DOX-induced systemic toxicity as well as modified pharmacokinetic pattern of the prepared targeted formulation in comparison with free DOX. It should be noted that the 5 mg/kg of DOX was found as the best-acceptable dose which injected intravenously in mice^33^. However, here we used 0.2 mg/kg of DOX. The aforementioned proper biodistribution (Fig. 7), the tumor inhibitory effects (Fig. 8a), and survival rate of SPION/PVD/MUC1_Apt_/DOX (Fig. 8b) suggested that this low dose of DOX can be the appropriate therapeutic dosage with slight toxicity when DOX directly intercalated within the aptamer sequence. Regarding the similarity between the tumor growth inhibition behavior of free DOX and non-targeted formulation, it could be ascribed to the partial accumulation of SPION/PVD/Scr_Apt_/DOX in tumor due to the EPR effect and thus showing tumor inhibitory effect similar to that of free DOX. In in vitro condition, free DOX enters the cell freely through passive diffusion, thereby inducing high toxicity in both target (C26) and non-target cells (CHO). On the contrary, in in vivo study, free DOX before reaching the tumor and entering the cell through diffusion, would be eliminated through renal clearance and liver metabolism thereby showing less anti-tumor activity in comparison with targeted formulation.

The biodistribution outcomes can be also ascribed to the decreasing of corona shielding around the Fe_3_O_4_ after PVD and MUC1_Apt_ conjugation on the surface of the targeted complex. It is well-accepted that when Fe_3_O_4_ NPs enter into relevant biological systems, their interaction with the plasma proteins results in the protein corona formation^25,44^. Protein coronas on nanomaterials surfaces can critically influence their target in recognition and internalization into the target cell. Another factor influencing the target cell uptake and elimination of nanomaterial-based complexes is emanated from the size of synthesized complexes^45^. The DOX-loaded SPION-based nanocarriers with size smaller than 200 nm escape from capturing by the reticuloendothelial system (RES), leading to the prolonged circulation time of formulations in blood-stream^46^ and better accumulation in tumors via the enhanced permeability and retention (EPR) effect due to increased tumor angiogenesis^47^. The obtained data verified that the prepared complex through the chelation of Fe_3_O_4_ NPs with PVD, despite its high PDI and heterogeneity, with size less than 200 nm, can safely be considered as an intravenous delivery system.

SPIONs are one of the US food and drug administration (FDA)-approved NPs that are successfully used as contrast agents in MRI. They can be easily functionalized for drug delivery, demonstrating great potential for theranostic applications^48,49^. The coating of SPION with organic materials can improve the colloidal stability which can facilitate the implementation of SPION as contrast agents for MRI. The MRI experiment revealed a noteworthy higher tumor accumulation of targeted SPION/PVD which can still be detected even 24 h post-injection (Fig. 6). These results are in agreement with enhanced cellular toxicity, the beneficial effects on inhibiting tumor, and higher survival rate of SPION/PVD/MUC1_Apt_/DOX versus SPION/PVD/Scr_Apt_/DOX because of its receptor-mediated endocytosis. All of these findings might be related to specific binding of MUC1_Apt_ to its overexpressed receptors (specific ligand-receptor interaction) in cancer cells, helping possibly a delay in extravasations from tumor tissues leading to the remain of the targeted formulation in the tumor site.

The efficiency of a contrast agent arise from its relaxivity, which is the proportionality constant of the measured rate of relaxation over a range of contrast agent concentrations^50^. The relaxivity relates to the magnetic properties of the contrast agent (including particle size, composition, and crystallinity), the molecular structure and kinetic of the prepared complex, as well as experimental conditions such as temperature, field strength, and the measurement media^50^. For example, Resovist® as an organ-specific MRI contrast agent, consists of carboxydextran-coated USPIO with predominantly results in a negative enhancement of normal liver parenchyma on both T2 and T1 weighted images^51^. Considering our previous studies^52,53^, in this study, a negative contrast (dark signal) was also obtained in both T1 and T2 weighted images of liver and tumor tissues after injection of formulation containing SPION. In general, T1 images of fat tissue (i.e. tumor tissue) exhibited a greater difference between tumor and SPIONs on T1 weighted images.

In conclusion, we provided a PVD-based theranostic nanocarrier for the targeted co-delivery of DOX as an anticancer drug and SPION as an imaging agent. MRI and flow cytometry assays confirmed the accumulation of designed nanoformulation in the tumor cells. Furthermore, the accumulation of MUC1_Apt_-targeted complex (SPION/PVD/MUC1_Apt_/DOX) was detected in tumor site even 24 h post-injection using ex vivo fluorescence imaging. The in vivo analysis revealed that the significant tumor inhibition and survival rate in the mice receiving a single-dose targeted-NPs in comparison with the non-targeted NPs. It could be concluded that SPION/PVD/MUC1_Apt_/DOX formulation provide an efficient dual-modality targeted NPs which could be employed as theranostic platform for the clinical cancer diagnosis and therapy.

## Materials and methods

### Materials

Iron (III) chloride hexahydrate (FeCl_3_.6H_2_O, 99%), iron (II) chloride tetrahydrate (FeCl_2_.4H_2_O, 99%), ammonium hydroxide (5 M), N-hydroxysuccinimide (NHS), 2,5-diphenyltetrazolium bromide (MTT), 1-ethyl-3-(3-dimethylaminopropyl) carbodiimide (EDC), 4-(2-hydroxyerhyl) piperazine-1-erhanesulfonicacid (HEPES), ethylenediaminetetraacetic acid (EDTA), SP Sepharose fast flow and Sephadex G-15 were purchased from Sigma Aldrich (Munich, Germany). Doxorubicin hydrochloride (DOX) was purchased from Euroasia Co., Ltd. (Delhi, India). GelRed (GR) (10,000X in water) was purchased from Biotium (USA). N50 neodymium magnet (50 × 50 × 30 mm) with 14 kilo Gauss remanence was purchased from Kaiven, Inc (China). All materials were of analytical grade. Cell culture media (RPMI1640), trypsin, fetal bovine sera (FBS), and penicillin/streptomycin solution were obtained from Gibco (Darmstadt, Germany). Chinese hamster ovary (CHO) and mouse colon carcinoma (C26) cell lines were achieved from the National Cell Bank of Iran, Pasteur Institute of Iran. MUC1 aptamer (MUC1_Apt_) and scrambled aptamer (Scr_Apt_) were obtained from Bioneer (Daejeon, South Korea) and Microsynth (Balgach, Switzerland) respectively, with the sequences as follow:

MUC1_Apt_: 5′- GCA GTT GAT CCT TTG GAT ACC CTG G–NH_2_–3′.

Scr_Apt_: 5′- AAT CAA CAT CAA TGT CTG AAG CGG ACT CCC C–NH_2_—3′.

All the method and protocols were performed in accordance with the guidelines and regulation of Mashhad University of Medical Sciences.

### Extraction, purification and characterization of pyoverdine

Pyoverdine was extracted from *P. fluorescens* IBRC-M 10752 strain (obtained from Iranian Biological Resource Center (IBRC), Microorganisms Bank Collections). Briefly, 100 µL of *P. fluorescens* suspension (~ 10^4^ CFU/mL) was inoculated into 500 mL Erlenmeyer flasks containing 200 mL of broth standard succinate medium (SSM/consisting of g/L: MgSO_4_, 7H_2_O, 0.2; KH_2_PO_4_, 3.0; K_2_HPO_4_, 6.0; (NH_4_)_2_SO_4_, 1.0; and succinic acid 4.0, pH 7.0.) and incubated at 28 °C with constant shaking at 120 rpm. After 72 h of incubation, the media were centrifuged at 10,000×*g* for 10 min at 25 °C and filtered via 0.22 µm membrane filter^54^. The cell-free supernatant was then acidified (pH: 2.5) and concentrated with ethyl acetate.

The dried PVD was resuspended in HEPES buffer (pH 7.0) and was passed through copper-chelated SP-Sepharose fast flow column (2.5 × 1.6 cm, 5 mL; GE Healthcare) to desalt and preliminary purify. After washing with HEPES buffer, the column was eluted with 20 mM acetate buffer (pH 4.0) and PVD-Cu containing fractions with the highest A_400_ absorbance were collected and lyophilized. Subsequently, the lyophilized residue was dissolved in 10 mM EDTA and was further purified by Sephadex G-15 column (1.5 × 80 cm; GE Healthcare), followed by ultrapure water elution and fractions with the highest fluorescence intensity (excitation/emission at 400/460 nm) were collected and lyophilized to obtain pure PVD^31,55^. The concentration of purified PVD was estimated based on A = εBC formula^56^ and characterized by using Arnow’s and Czsaky’s methods for catecholate and hydroxamate groups assay, respectively^57^. The purified PVD was also further analyzed by determination of λ_max_, fluorescence excitation/emission (400/460 nm), iron-chelation properties (fluorescence quenching of PVD at 460 nm by FeCl_3_ solution), FTIR in the range of 4000–400 cm^−1^, thin layer chromatography (TLC) and LC/MS/MS spectroscopy (AB SCIEX, Darmstadt, Germany)^57–59^.

### Synthesis of SPIONs and conjugation to PVD

The synthesis of SPIONs was performed using a co-precipitation method as previously described^60^. In deionized/deoxygenated water with nitrogen (N_2_), a mixture of Fe (III) chloride (0.1 M) and Fe (II) chloride (0.1 M) with the molar ratio 1:2 was prepared for 15 min. 3 mL NH_4_OH solution (5 M) was added slowly to this solution while stirred under N_2_ atmosphere at 25 °C until a deep black color appeared (~ 15–20 min). Another half-hour, the suspension was constantly stirred at 85 °C. Lastly, the ammonia was vaporized and an external magnetic field was applied to separate the black color precipitate, and freeze-dried after washing with double-distilled water (ddH_2_O). In the next step, a solution containing 2 mg/mL of both SPIONs and PVD was sonicated in an ultrasonic bath for 20 min at room temperature. Then, the mixture was centrifuged at 14,000×*g* for 10 min at 4 °C to further remove very large aggregates.

The particle size distribution, polydispersity index (PDI) and zeta potential of SPIONs and SPION/PVD conjugates were assayed by dynamic light scattering (DLS) ZetaSizer (NANO-ZS, Malvern, UK). Field emission scanning electron microscope (FE-SEM, TESCAN MIRA3, Brno, Czech Republic) was applied to evaluate the morphology, particle size and EDX pattern of complexes. The magnetic properties of SPION and SPION/PVD conjugates were assessed by the vibrating sample magnetometer (VSM) to evaluate the Magnetic field dependence at room temperature under circulate magnetic field in the range of − 20,000 up to 20,000 Oe. The thermal stability and the content of conjugated PVD to SPION, was investigated by the thermogravimetric analysis (TGA) method under nitrogen in the temperature range 50–600 °C with a heating rate of 15 °C/min. The FT-IR spectra of PVD and SPION-PVD were also performed by Nicolet Avatar 360 FTIR spectrometer (Thermo Nicolet Corp, USA) in the range of 400–4000 cm^−1^. The SPION-PVD conjugates were also further analyzed by fluorescence quenching at 460 nm.

### Conjugation of MUC1_Apt_ to SPION/PVD

The NH_2_-MUC1_Apt_ molecules were covalently linked to the exposed carboxyl group (–COOH) of PVD in SPION/PVD complex using the EDC/NHS reaction^61^. Briefly, a suspension of SPION/PVD (2 mg/mL) in 980 µL phosphate-buffered saline (10 mM, pH 7.4) was adjacent with an excess of EDC (5 mg/mL) and NHS (3 mg/mL) for 1 h at room temperature to activate the terminal carboxyl group on SPION/PVD. After incubation, 20 µL of NH_2_-MUC1_Apt_ (10 μM) was supplemented to the reaction medium and stirred for 12 h at 25 °C. Then, the mixture was concentrated to 100 µL by centrifugation (12,000×*g*, 10 min, 4 °C). The formed conjugate was magnetically collected from the system and washed twice with DNase/RNase-free water to remove the reactants and finally was re-suspended in 100 µL PBS. To confirm the formation of conjugates, 2.5% agarose gel electrophoresis in TBE buffer for 25 min at 90 mV was applied. Besides, in order to investigate the attachment of MUC1_Apt_ to SPION/PVD, the formed conjugate was treated with Dithiothreitol (DTT) as a reducing solution and then run on the gel. The SPION/PVD/MUC1_Apt_ size, ζ potential, PDI, and morphology were also investigated using Zetasizer and FE-SE.

### DOX loading in SPION/PVD/MUC1_Apt_ complexes

DOX-loaded SPION/PVD/MUC1_Apt_ was formulated by the addition of constant DOX concentration (2 µM) to SPION/PVD/MUC1_Apt_ formulations with 0, 2, 4, 6, 8 and 10 µM MUC1 aptamer content, respectively. After incubation for 4 h at room temperature, the loaded complexes were magnetically removed from the suspension and the supernatants were collected. The DOX content of the supernatants was determined according to the calibration curve of DOX concentration via monitoring of fluorescence intensity of DOX by a microplate reader (excitation/emission 480/595 nm). Lastly, DOX entrapment efficiency (EE) and the drug loading content (LC) of SPION/PVD/MUC1_Apt_ were determined based on the following equations^33,62^.

$${\text{EE}}\;~\left( \% \right) = ~\frac{{({\text{Mass}}\;~{\text{of}}\;{\text{~DOX}}\;~{\text{initially}}\;~{\text{used}} - {\text{Mass}}\;{\text{~of}}\;{\text{~supernatant}}\;~{\text{DOX}})}}{{{\text{Initial}}\;~{\text{amount}}\;~{\text{of}}\;{\text{feeding}}\;{\text{~DOX}}}}~ \times 100$$

$${\text{LC}}~\;\left( \% \right) = ~\frac{{{\text{Mass}}\;~{\text{of}}\;~{\text{loaded}}\;~{\text{DOX}}~\;{\text{in}}\;~{\text{the}}~\;{\text{formulation}}}}{{{\text{Mass}}\;{\text{of}}\;{\text{final}}\;{\text{formulation}}}}~ \times 100$$

The size, ζ potential, PDI and morphology of SPION/PVD/MUC1_Apt_/DOX (final formulation) were also investigated using Zetasizer and FE-SE.

### In vitro DOX release

The in vitro release pattern of DOX was accomplished in either PBS at pH 7.4 and citrate buffer at pH 5.5. SPION/PVD/MUC1_Apt_/DOX complex was resuspended in 400 µL PBS and citrate buffer and incubated at 37 °C with shaking at a speed of 80 rpm. For estimation of the released DOX, the complex was magnetically collected and the supernatant was taken at 0, 3, 6, 12, 24, 36, 48, 72 and 96 h to measure the released DOX spectrofluorimetrically (excitation/emission at 480/595 nm). At predetermined time intervals, the drawn media was replaced by the same amount of fresh buffer. The following equation was applied to estimate the DOX accumulative release percentage (AR%)^62^.

$${\text{AR~}}\;\left( \% \right) = ~\frac{{{\text{Concentration}}\;{\text{of}}\;~{\text{released}}\;~{\text{DOX}}}}{{{\text{Concentration}}\;{\text{of}}~\;{\text{loaded}}\;~{\text{DOX}}~\;{\text{in}}~\;{\text{the}}~\;{\text{formulation}}}}~ \times 100$$

### Cell viability assay of the synthesized formulations

According to the results of a dose-escalating experiment, IC_50_ of DOX was obtained 0.15 and 0.5 μM for C26 and CHO cells, respectively. The cytotoxicity of free DOX, SPION/PVD/MUC1_Apt_ and SPION/PVD/MUC1_Apt_/DOX with equivalent concentration of 0.15 μM DOX for C26 and 0.5 μM DOX for CHO cells, was investigated using MTT assay. A DOX-loaded scrambled aptamer (Scr_Apt_) which conjugated to SPION/PVD was also used as a non-targeted complex (SPION/PVD/Scr_Apt_/DOX). 5 × 10^3^ of C26 and CHO cells were seeded onto 96-well plates for 24 h and then treated with the above-mentioned formulations for 4 h. Then, the culture media were exchanged with fresh medium and incubated at 37 °C and CO_2_ 5% for 48 h. Then, a further incubation (~ 4 h) was carried out after adding 20 μL of MTT solution (5 mg/mL in PBS) to each well. Then, MTT was replaced with 100 μL of DMSO to dissolve formazan crystals at room temperature. The optical density was detected at 570 and 630 nm using a microplate reader (BioTeK, USA)^33^. Furthermore, a competitive assay was also done to verify the selective targeting property of MUC1 by the addition of an excessive amount of free MUC1_Apt_ 30 min before the adding of SPION/PVD/MUC1_Apt_/DOX to the wells^33^. Viability (%) was compared with untreated cells according to data of three individual assessments.

### Cellular uptake level of synthesized formulations

C26 and CHO cells, as respective MUC1 positive and MUC1 negative control, were seeded in 12-well plates at density of 1 × 10^5^ cells/well and cultured in RPMI 1640 containing 10% FBS and 1% penicillin/streptomycin and kept 37 °C in a humidified incubator with 5% CO_2_. After 24 h, the cells were treated with free DOX, SPION/PVD/MUC1_Apt_/DOX and SPION/PVD/Scr_Apt_/DOX (at DOX equivalent concentration 1 μM for C26 and CHO) for 2.5 h^33,35,63^. To survey the targeting efficiency of the SPION/PVD/MUC1_Apt_/DOX, a competitive assay was also designed, in which C26 and CHO cells were incubated with an excess amount of free MUC1_Apt_ 30 min before the addition of SPION/PVD/MUC1_Apt_/DOX. To perform fluorescence microscopy imaging, the media were discarded and replaced with fresh media and the appropriate pictures were accordingly taken. For flow cytometry study, after removing of media and washing twice, the cells were trypsinized and the cells suspension in non-FBS media was centrifuged at 1600 rpm for 5 min. Afterward, the pellet was dissolved in cold PBS (pH 7.4) and the fluorescence intensity was measured using a BD FACSCalibur™ in the FL2 channel and analyzed by FlowJo 10.6 software.

### In vivo antitumor efficacy of the synthesized formulations

BALB/c mice were obtained from Animal Resources Center (Pasteur Institute, Iran). To obtain tumor-bearing mice, C26 cells (3 × 10^5^ cells/100 μl PBS) were inoculated into the right side subcutaneous region of male 4–5 week old BALB/c (~ 20 g) mice. When the size of the tumor reached ~ 50 mm^3^, the mice were randomly divided into five groups (n = 5). Group I (control group) was injected with 200 μL PBS, groups II, III, and IV were intravenously injected with 200 μL of free DOX, SPION/PVD/MUC1_Apt_/DOX and SPION/PVD/Scr_Apt_/DOX (DOX equivalent concentration 0.2 mg/kg) via a single tail-vein injection. The mice body weight and survival rates of the tumor-bearing mice were investigated and the tumor volume was calculated by the formula length × width × height × 0.5^33,35^. The mice were checked 26 days after tumor induction until they reached endpoint (body weight loss of > 20%).

### Biodistribution study of the synthesized formulations

When the size of the tumor of BALB/c mice reached 200–300 mm^3^, the mice were intravenously injected with free DOX, SPION/PVD/MUC1_Apt_/DOX and SPION/PVD/Scr_Apt_/DOX (DOX equivalent concentration 0.2 mg/kg). After 6 and 24 h of injection, the animals were sacrificed and tumor tissues, heart, lungs, liver, kidneys, and spleen were excised and biodistribution pattern was measured according to the fluorescent intensity of these organs at λ_ex_ = 450 and λ_em_ = 580 by a Kodak FX Pro in vivo imaging system.

### MRI

MRI technique was used to visualize the tumor accumulation of non-targeted NPs (SPION/PVD) and SPION/PVD/MUC1_Apt_/DOX as a theranostic platform. BALB/c mice with 200–300 mm^3^ C26 tumor size, were administered with a 200 μl of these formulation containing an equivalent concentration of 0.6 mg/mL SPION and a DOX concentration 0.2 mg/kg in targeted-NPs via a single tail vein injection. For MRI, a intraperitoneal inoculation of ketamine/xylazine combination with respective 80 and 10 mg/kg body weight was used to anesthetize the mice. The T1-weighted images from coronal plane of C26 tumor-bearing mice were obtained using a 1.5 T MRI scanner (MAGNETOM Symphony; SIEMENS, Germany) at pre-injection, 6 and 24 h post-injection of formulations. The following parameters were applied for T1-weighted scans: time of echo (TE) = 11 ms; time of retention (TR) = 453 ms; slice thickness = 2.5 mm and resolution = 256 pixel.

### Statistical analysis

The statistical analysis was performed by one-way analysis of variance (ANOVA) to determine the significant difference between groups (a P-value less than 0.05) by using GraphPad Prism Version 8.0 (GraphPad Software Inc, USA, https://www.graphpad.com). Quantitative results are indicated as the mean ± standard deviation (SD).

### Ethics declarations

All animal experiments were approved by the Institutional Ethical Committee and Research Advisory Committee of Mashhad University of Medical Sciences guidelines under registration number (IR.MUMS.REC.950835). The study was also carried out in compliance with the ARRIVE guidelines^64^.

## Supplementary Information

Supplementary Information.
