# Supplementary material for: Targeted SPION siderophore conjugate loaded with doxorubicin as a theranostic agent for imaging and treatment of colon carcinoma
Source: Sci Rep. 2021 Jun 22;11:13065. doi: 10.1038/s41598-021-92391-w (PMC8219724; doi:10.1038/s41598-021-92391-w)
Supplement: Supplementary file 1 — Supplementary Information. [file 41598_2021_92391_MOESM1_ESM.pdf]

Supplementary information

Targeted SPION siderophore conjugate loaded with doxorubicin as a theranostic agent for imaging and treatment of colon carcinoma

Rahim Nosrati[1], Khalil Abnous[2,3], Mona Alibolandi[2], Jafar Mosafer[4,5], Sadegh Dehghani[6], Seyed Mohammad Taghdisi[7]*, Mohammad Ramezani[2]**

[1] Department of Pharmaceutical Biotechnology, School of Pharmacy, Mashhad University of Medical Sciences, Mashhad, Iran
[2] Pharmaceutical Research Center, Pharmaceutical Technology Institute, Mashhad University of Medical Sciences, Mashhad, Iran
[3] Department of Medicinal Chemistry, School of Pharmacy, Mashhad University of Medical Sciences, Mashhad, Iran
[4] Department of Nanomedicine, School of Paramedical Sciences, Torbat Heydariyeh University of Medical Sciences, Torbat Heydariyeh, Iran
[5] Department of Radiology, 9 Dey Educational Hospital, Torbat Heydariyeh University of Medical Sciences, Torbat Heydariyeh, Iran
[6] Department of Medical Biotechnology and Nanotechnology, Faculty of Medicine, Mashhad University of Medical Sciences, Mashhad, Iran
[7] Targeted Drug Delivery Research Center, Pharmaceutical Technology Institute, Mashhad University of Medical Sciences, Mashhad, Iran

Corresponding authors:
Mohammad Ramezani, Pharm. D, Ph.D.
Seyed Mohammad Taghdisi, Pharm. D, Ph.D.
School of Pharmacy, Pharmaceutical Research Center,
Mashhad University of Medical Sciences, Mashhad, Iran
Tel: +98-5131801201
Fax: +98-5138823251
Email: Ramezanim@mums.ac.ir; Taghdisihm@mums.ac.ir

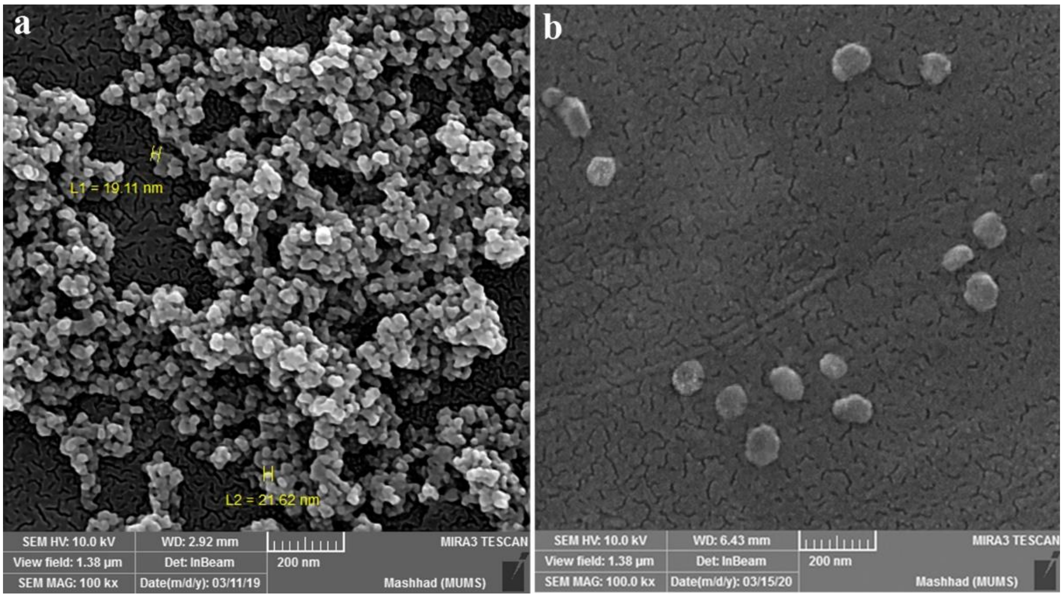

Figure S1. SEM images of (A) bare SPION and (B) SPION/PVD/MUC1$_{Apt}$/DOX system.

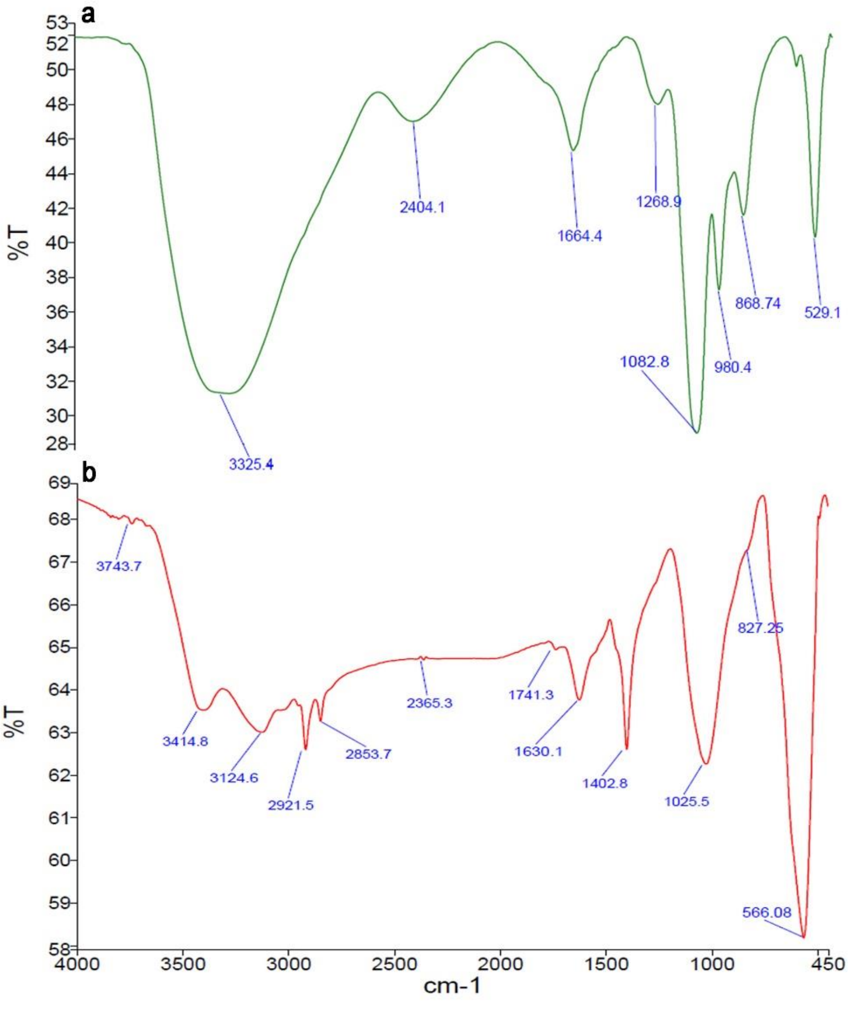

Figure S2. FTIR absorption spectra of (a) the PVD and (b) SPION/PVD

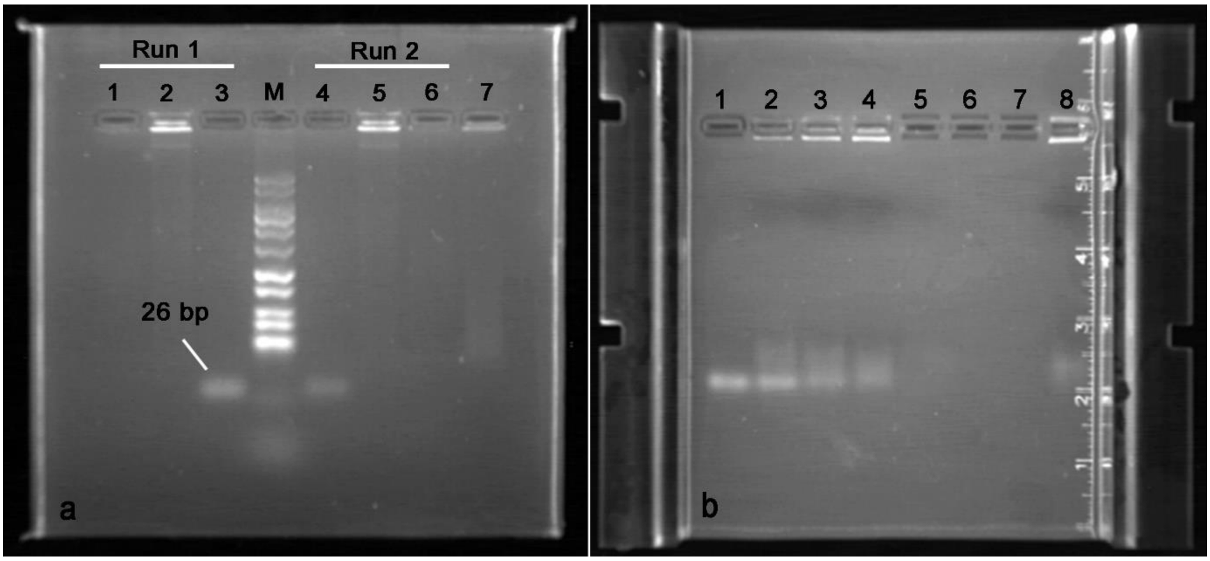

Figure S3. a) Gel retardation assay for characterization of SPION/PVD attachment to $MUC1_{Apt}$. SPION/PVD complex (Lane 1) did not show any band, SPION/PVD/$MUC1_{Apt}$ conjugate (Lane 2) did not migrate through the gel, while free $MUC1_{Apt}$ (Lane 3) moved through it and the band was observable in its correct place according to the 50 kb ladder (Lane M). Lanes 4, 5, and 6 show the repeated test of free $MUC1_{Apt}$, SPION/PVD/$MUC1_{Apt}$, and SPION/PVD, respectively. Lane 7 shows a sample of $MUC1_{Apt}$ attached complex performing at 6 h instead to 12 h; b) In the presence of DTT, $MUC1_{Apt}$ was cleaved from SPION/PVD/$MUC1_{Apt}$ conjugate depending on the DTT concentration and moved through the gel (lanes 2, 3, and 4). Lane 1 shows free $MUC1_{Apt}$ in the gel electrophoresis. In Lanes 5, 6, and 7 no sample was run while Lane 8 shows SPION/PVD/$MUC1_{Apt}$ without DTT treatment.

Table S1: Stability analysis of the prepared formulation.

	Bare SPION		SPION/PVD/$MUC1_{Apt}$/DOX	
Time (h)	Size (nm)	PDI	Size (nm)	PDI
2	69.7±6.8	0.24±0.04	131.2±6.2	0.39±0.04
6	178.7±16.3	0.36±0.01	128.8±3.2	0.36±0.01
12	307.3±13.6	0.45±0.06	129.3±1.5	0.27±0.06
24	359.7±8.9	0.41±0.03	126±4.7	0.32±0.02
